# Addressing the Dangerous Consequences of the Resurgence of Measles and Rubella: The Critical Need for a Global Target

**DOI:** 10.3390/vaccines12060698

**Published:** 2024-06-20

**Authors:** Jon Kim Andrus

**Affiliations:** Department of Global Health, Milken Institute of Public Health, George Washington University, Washington, DC 20052, USA; andrus@gwu.edu

I am delighted and honored to be Guest Editor of this *Vaccines* Special Issue on measles and rubella elimination. I was extremely fortunate to have a team of co-editors who assisted with this work. Their experience in the field of immunization, and with the elimination of measles, rubella, congenital rubella syndrome is noteworthy. As evidenced by their biographies, the collective sum of their knowledge and experience is staggering.

On behalf of all the co-editors—Sunil Bahl, David Durrheim, Katrina Kretsinger, Mark Papania, Susan Reef, Paul Rota, and Peter Strebel—I would like to extend my sincerest thanks and appreciation to all the authors of the manuscripts included in this supplement. We are fortunate to have the support of *Vaccines* in publishing this Special Issue, a journal that promotes free and open access to science and boasts a considerable reader rating as a result. In addition to our Editorial Team reviewing the manuscripts, each article underwent another two independent peer review evaluations to be accepted.

I would like to extend special thanks to Xinya Huang and Sydnee Pang from the editorial staff of *Vaccines*, as well as Mark Papania and Amanda McKinnon from CDC, Atlanta, USA, all of whom provided invaluable administrative support. Most importantly, we should to acknowledge all the dedicated government workers, stakeholders, and field workers working for a world free of measles, rubella, and congenital rubella syndrome. I hope you enjoy reading this supplement as much as we enjoyed collating it.

However, this gratitude is dampened by an uneasy feeling of foreboding. This “sense” of gloom is linked to an incredible opportunity missed, stemming from my own personal experience. In the field of public health, a “story” can potentially confer just as much information as a dataset or statistical analysis. With this in mind, please bear with me as I briefly share mine. 

My first assignment overseas was in 1985, serving as a Peace Corps volunteer as the District Medical Officer of the Mchinji District Hospital in Malawi, Africa. The 66-bed hospital I supervised as the only doctor in the District had a measles ward that overflowed with children at the start of every annual measles season. With too few beds to meet this overwhelming demand, two or three children were assigned to every bed. Families filled the hallways and were scattered under beds. Mothers mourned the daily death toll, wailing terribly. The noise was overwhelming. Some of the children had peeling skin due to severe protein malnutrition, a foreboding sign. Their fate was an increased risk of death, pneumonia, encephalitis, and blindness. These painful memories of all the deaths and co-morbidities that could have been prevented are forever etched into my soul.

Returning on a World Health Organization (WHO) assignment to Mchinji some 15 years later, after Malawi had conducted a nationwide measles catch-up vaccination campaign, I became convinced that measles could be eliminated, even in some of the most direly affected places in the world. On revisiting during the typical measles season, the ward had been shut down. No more admissions, no more deaths, no more suffering from this dreaded disease. Such was the impact of the catch-up campaign and the consolidation of immunization services implemented here. The catch-up campaign treated every child aged <15 years with a measles containing vaccine (MCV). As described, the results were nothing short of phenomenal.

Unfortunately, there is a caveat to this story. Without follow-up vaccination campaigns being conducted every four years (targeting children aged <5 years with a measles vaccine), measles undoubtedly returns, even despite comparatively high levels of routine coverage. This has indeed played out in Malawi and in other countries in Africa, an oversight requiring swift correction, along with upgrading essential immunization systems, improving the vaccination coverage for other antigens, and optimizing field surveillance.

In absorbing the following research, I hope you share in my sincere belief that the voice of reason resoundingly maintains that global measles rubella elimination (MRE) is feasible, possible, and sustainable [1]. Many, including me, would argue that MRE is a moral imperative [2]. How can we not eliminate a disease so devastating and yet known to be preventable using existing strategies? The measle antigen in current use averts more deaths than any other antigen, and a combined measles–rubella vaccine could also eliminate the leading cause of congenital birth defects due to infectious disease. A global target is required, as pertinently stipulated by all the Chairs of the WHO Regional Verification Commission in their article. No more missed opportunities of this kind can occur. As you read, reflect on the fact that the past two decades of remarkable global progress are at risk of becoming redundant as measles infection reemerges and surges. The question is whether a renewed global commitment to protecting children around the world will be made. 
